# Neighborhood Socioeconomic Disadvantage and Health Status Among African Americans Living in Low-Income Housing

**DOI:** 10.1093/geroni/igaa057.1921

**Published:** 2020-12-16

**Authors:** Adrienne Aiken-Morgan, Dextiny McCain, Karon Phillips, Keith Whitfield

**Affiliations:** 1 North Carolina A&T State University, Greensboro, North Carolina, United States; 2 North Carolina A&T State University, Kernersville, North Carolina, United States; 3 UMBC, Baltimore, Maryland, United States; 4 Wayne State University, Detroit, Michigan, United States

## Abstract

Research has shown the importance of social determinants of health in explaining racial/ethnic disparities in many health outcomes; however, less attention has been given to within-group differences in social determinants of health among low-income African American older adults. The Physical and Cognitive Health Pilot Study (n=50) was utilized to examine associations between level of neighborhood socioeconomic disadvantage and self-reported health in African American older adults living in public housing in Durham, NC and Annapolis, MD. Results from ANOVA showed that Durham participants living in more disadvantaged neighborhoods had statistically significantly worse cardiovascular health, higher depression symptoms, worse sleep quality, and higher alcohol use (p=.05) than Annapolis participants living in a more resource-rich neighborhood. These findings suggest that among low-income African American elders, greater neighborhood/state socioeconomic disadvantage is associated with worse health status. Future research should consider neighborhood context as an essential variable when assessing health status among aging African Americans.

